# A Comparison of Experimental and Theoretical Relations Between Young’s Modulus and the Flexural and Longitudinal Resonance Frequencies of Uniform Bars

**DOI:** 10.6028/jres.064A.014

**Published:** 1960-04-01

**Authors:** S. Spinner, T. W. Reichard, W. E. Tefft

## Abstract

The relations from which Young’s modulus may be computed from mechanical flexural and longitudinal resonance frequencies have been established by an empirical method using two sets of steel bars. Both sets contained rectangular and cylindrical specimens. For longitudinal vibration of cylindrical specimens, the agreement between the empirical curves and Bancroft’s corresponding theoretical relation was within experimental error if Poisson’s ratio for both sets is taken to be 0.292. For flexural vibrations, the agreement between the empirical curve and the corresponding theoretical relation developed by Pickett is also within experimental error for about the same value of Poisson’s ratio for the rectangular specimens of both sets; but for cylindrical specimens, the empirical values are somewhat lower than those predicted by the theory.

## 1. Introduction

In a previous paper, [1]^1^ an empirical relation was established from which the shear modulus could be calculated from the torsional resonance frequency using uniform steel bars of different rectangular cross sections. The empirical relation was compared with corresponding theoretical approximations. The purpose of the present paper is to establish similar relations from which Young’s modulus may be determined from the flexural and longitudinal mechanical resonance frequencies for bars of round and rectangular cross section. These empirical relations are also compared with corresponding theoretical equations when feasible.

As in the previous work, advantage is taken of the fact that these resonance frequencies can be determined to an accuracy which, when combined with comparable accuracy of dimensions, is sufficient to yield empirical results good to four significant figures.

In fact, it is this increased accuracy to which modern experimental techniques have advanced dynamic elastic measurements that has made it possible to check in a more precise manner the theoretical results of such analysts as Rayleigh, Kelvin, Poisson, and Stokes [2].

As usually happens in such cases, this increased experimental accuracy has, in turn, led to a refinement and extension of the theory. Some equations had lain dormant for many years because, though presumably “complete” and “correct,” they were nevertheless expressed in so general a form that numerical solutions for most real cases were too cumbersome to be of practical value. Such equations have recently been solved for given boundary conditions. These solutions have often taken advantage of modern computing devices. A particular case in point is the set of Pochhammer-Chree equations, relating the most general case of elastic waves in rods to their elastic moduli. These equations, almost forgotten for more than 50 years, were solved by Bancroft [3] for the case of longitudinal waves and by Hudson [4] for flexural waves. A recent article by Davies [5] presents a comprehensive review and bibliography of the advances in this field up to the present time.

For Young’s modulus, the problem of establishing an empirical relation is complicated by the fact that the cross sectional correction for both flexural and longitudinal vibrations depends upon Poisson’s ratio as well as the dimensions. This is in contrast with shear for which the cross sectional correction is independent of Poisson’s ratio. Consequently, the results to be presented here are more limited than those previously given for shear since these results apply mainly to those materials having Poisson’s ratios approximately equal to those used here. Furthermore, when comparing the empirical with theoretical relations, any error in the value of Poisson’s ratio assigned to the specimens would result in a corresponding error in the comparison of the correction factors. This error would increase as the ratio of cross section to length increased.

## 2. Experimental Procedures

### 2.1. General

The basic experimental approach consists in determining the flexural and longitudinal resonance frequencies of specimens of known mass and dimensions, and assuming their uniformity of Young’s modulus and density, to derive the empirical relation needed for the determination of Young’s modulus from the mechanical resonance frequency. Data to be presented later will show that the assumption of uniform density and Young’s modulus is quite justified.

### 2.2. Specimens

Two separate sets of steel specimens were used in this investigation. Each set of specimens was cut from its own parent piece to insure the greatest possible uniformity of intrinsic Young’s modulus and density from specimen to specimen within each of the two sets.

One source was a cylindrical bar of cold drawn steel about 1 in. in diameter, designated as SAE No. 1020. Originally 18 specimens, I–5 through I–22, ranging in length from about 3 to 12 in. were cut from the parent stock. Subsequently some of these rods were further shortened or machined to square cross section to extend the range of the experimental data. All of the specimens from this source are henceforth classified as set I. Exact dimensions and other related data for set I are given in table 1.

The other source of specimens was a bar of 2-in. square stock of hot rolled and annealed tool steel designated by the trade name “Stentor.” The original specimens from this source were the same 12 pieces (II–1 through II–12) of equal lengths but different rectangular cross sections that were used in the investigation for shear modulus [1]. As for set I, some of these specimens were further reduced in dimensions or machined to circular cross section. All specimens from the second source are classified as set II and data for these specimens are given in table 2.

The dimensions of both sets of specimens are accurate to ±.001 cm. The density was calculated from the mass and the dimensions of the specimens. The average density for the specimens of set I was 7.851 g/cm^3^, and that of set II was, as previously given [1], 7.814 g/cm^3^. The standard deviation of this measurement was 0.002 g/cm^3^ for both sets. This small variation in density is good evidence for the intrinsic uniformity of the specimens of each set. Although the density variations are within the error of the measurement, the mass and dimensions of each particular specimen were used in most calculations rather than the average value of density. The density of some randomly selected specimens of both sets was also checked by weighing in air and while immersed in liquid and was found to agree with the above values within the error of their determination.

Actually, for the specimens of set II, the density, *ρ*, by the immersion technique was found to be 7.816 g/cm^3^. Subsequent determination of *ρ*, calculated from the mass and volume of two specimens machined to a higher degree of accuracy than the others (specimens II–4*r*_1_ and II–4*r*_2_), agreed with the value obtained by immersion and is believed to be the most reliable value for the specimens of set II.

### 2.3. Method

The mechanical longitudinal and flexural resonance frequencies of both sets of specimens were determined by the dynamic method previously described [1]. Briefly, one of the mechanical resonance frequencies of the specimen is excited by an electromagnetic driver. The increased amplitude of vibration of the specimen at resonance is detected by a crystal pickup whose output, together with a signal of the same frequency, produces a Lissajou pattern on a cathode-ray oscilloscope. The different types of vibrations are obtained by appropriate placement of the driver and pickup with respect to the specimen.

As with torsional vibrations the longitudinal and flexural resonance frequencies were excited and detected by more than one method.

In the first method the specimens were supported on foam rubber in the vicinity of the nodal points and driven through air by a tweeter type driver. A crystal pickup placed lightly against the proper part of the specimen detected the vibrations. Both longitudinal and flexural vibrations could be obtained by this method.

The second method could be used only to obtain flexural vibrations and was most appropriate for the lighter specimens. This method consisted in suspending the specimens from two cotton fibers, one fiber being attached to a phonograph record cutting head as the driver and the other fiber being attached to a crystal pickup. Unlike the case for torsion, it was not necessary for the points of suspension to be at opposite faces of the specimen.

A third method, combining certain features of the first two, consisted in suspending the specimens from two cotton fibers as in the second method but driving them through air with a tweeter and detecting the vibrations with a crystal pickup as in the first method. This third method could be used to obtain both flexural and longitudinal vibrations and was satisfactory for heavy as well as light specimens. The highest resonance frequencies could be obtained most readily by this method.

The accuracy of the resonance frequencies obtained by the last two methods was usually somewhat better than that obtained by the first method. However, by any of these methods, the accuracy of the resonance frequencies was usually better than 1 part in 4,000 [1].

The fundamental longitudinal and flexural resonance frequencies for the specimens of sets I and II are given respectively in tables 1 and 2. Inasmuch as the specimens of set II are rectangular in cross section, two separate flexural resonance frequencies occur about both longitudinal planes of symmetry (flatwise and edgewise). The fact that the edgewise flexural frequency is the same for specimens II–2 through II–12 is of considerable significance as will be explained. Table 3 gives frequencies of overtones of longitudinal resonance vibrations of four specimens of set I and one specimen of set II.

It may be observed that longitudinal resonance frequencies of over 50,000 cps are recorded for both fundamentals and overtones. These remarkably high values of resonance frequency which can be excited and detected by a method sometimes described as “sonic,” are explained by the nature of the specimens and also by the fact that the response of both driver and pickup, though reduced, persists at frequencies considerably above their rated upper frequency limit. This reduced response is amplified and detected as a recognizable pattern on the scope.

Since the upper frequency response obtained is more than 2½ times higher than the nominal upper limit of the sonic range and will probably go higher as experimental techniques improve, it is felt that the term, “mechanical resonance methods,” would be more appropriate than “sonic methods” to describe the experimental procedure used.

## 3. Calculations, Results, and Discussion

### 3.1. Longitudinal Resonance Frequencies for Cylindrical Specimens of Sets I and II

The following relations for this type of vibration are recalled. First, a rod vibrating in this manner satisfies the condition that

l=nλ/2 (1)

where *l*=length of specimen, *n*=order of resonance frequency. For the fundamental, *n* = 1, for the first overtone *n*=2, etc., λ=wavelength of the vibration in the specimen. This leads to the well-known relation between the velocity of the longitudinal wave, *v*, and the longitudinal resonance frequency, *f_n_*,

$$v=\frac{2lf_{n}}{n}.$$ (2)

The subscript after the *f* indicates the order of the resonance frequency.

For an infinitely thin specimen of length *l*, *v* becomes the “rod velocity,” *v*_0_. Rayleigh’s familiar approximation, given below, shows the amount by which *v*, in a specimen of finite circular cross section is reduced from *v*_0_.

$$v=v_{0}{\left[1+{\left(\frac{\pi n\mu r}{2l}\right)}^{2}\right]}^{-1}$$ (3)

where *μ*=Poisson’s ratio and *r*=radius of the rod.

The relation showing the effect of cross section is often expressed in terms of *v/v*_0_ as a function of *D/λ*, *D* being the diameter of cross section. This convention will be followed here. From eq (1) it is seen that *D*/λ=*nr/l*.

Empirical values of *v/v*_0_ can be conveniently calculated after the value of *v*_0_ has been determined. We consider first the specimens of set I. Substitution of the appropriate values for the longest specimen, I–22, (having the lowest *r*/*l*) in eq (3) shows that *v*=0.9996*v*_0_. Specimens I–19, I–20, and I–21 are sufficiently long to give an average for the ratio, *v/v*_0_, from eq (3) equal to that for specimen I–22. At the low values of *r*/*l* associated with these specimens, the choice of a proper value of *μ* is no problem, since any reasonable variation about the selected value of 0.3 (say from 0.26 to 0.32) will have only a negligible effect on the result. Also, at such low values of *r*/*l* any difference between Rayleigh’s and corresponding equations, such as Bancroft’s, will also be negligible. Therefore, the average value of *v*_0_=5152 m/sec, obtained by substituting the values for these four longest specimens in eq (2) and dividing by 0.9996, is taken to be the rod velocity for the specimens of set I. The empirical values of *v/v*_0_ for the remainder of the specimens of set I are then found from the following equation

$$v/v_{0}=\frac{2lf_{n}}{5152n}.$$ (4)

**Figure 1:**
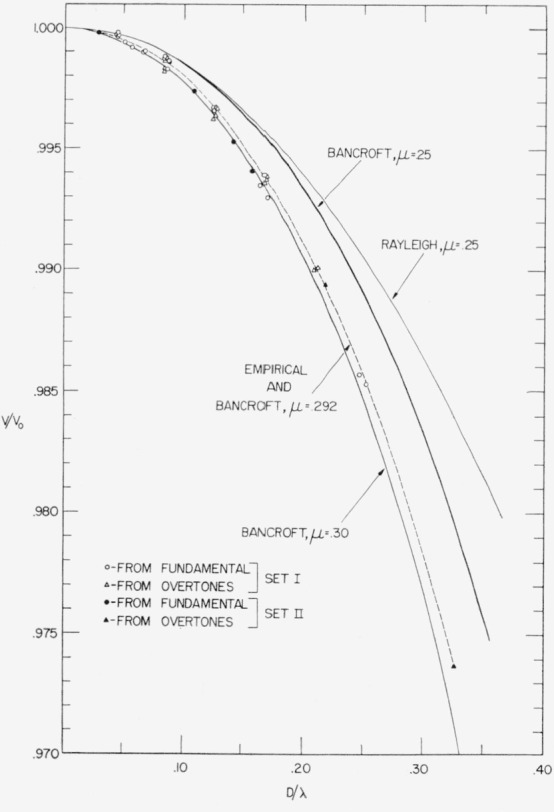
Effect of the ratio of diameter (*D*) to wavelength (*λ*) on the ratio of the velocity of the longitudinal wave (v) to the rod velocity (v_0_) for two sets of cylindrical steel specimens. Theoretical curves are included for comparison.

These *v/v*_0_ values are given numerically in table 1 and graphically, as a function of *D/*λ in figure 1.

The empirical values of *v/v*_0_ for the specimens of set II are found in the same manner. The reference specimens in this case, having the lowest value of *r*/*l*, were II–4*r*_1_ and II–4*r*_2_. These are recalled to be the specimens that were more accurately machined. The purpose of this was to obtain a more reliable base value. For these specimens, *v/v*_0_=0.9996, *v*_0_=5199 m/sec, and empirical values for the other specimens are found from the equation

$$v/v_{0}=\frac{2lf_{n}}{5199n}.$$ (4a)

Numerical values of *v/v*_0_ for these specimens are given in table 2 and are plotted along with those of set I in figure 1.

For both sets of specimens, *v/v*_0_ for higher values of *D*/λ was obtained both by vibrating the shortest specimens at their fundamental resonance frequency and also (since D/λ=*n r*/*l*) by vibrating some specimens at higher overtones. The data for these overtones are found in table 3.

It is observed from figure 1 that not only do the empirical points fall on the same curve, within experimental error, whether determined from the fundamental or overtones of either set of specimens, but also the points for both sets of specimens also fall on this same curve.

Since *v*_0_ and the density, *ρ*, are known, Young’s modulus, *E*, can be determined for each set of specimens from the equation,

$$E={v_{0}}^{2}\;\rho$$ (5)

for set I, *E*=2084×10^9^ dynes/cm^2^=2084 kilobars, and for set II, *E*=2113 kilobars.

Bancroft’s [3] numerical solution of the Pochhammer-Chree equation for longitudinal waves has already been mentioned. His values for *μ*=0.25 and *μ*=0.30 are plotted, along with values based on Rayleigh’s equation for *μ*=0.25, in figure 1. Bancroft’s solution is seen to reduce *v/v*_0_ by a greater amount than Rayleigh’s for a given value of *μ*. Since Bancroft’s solution is considered more exact than Rayleigh’s, comparison of the empirical points will be made with Bancroft. Graphical interpolation between Bancroft’s values for *μ* = 0.25 and *μ*=0.30 at D/λ=0.25 shows the empirical curve to agree with Bancroft for the case where *μ*=0.292. That is, if the *μ* of the specimens of sets I and II is 0.292, then agreement of the empirical with Bancroft’s solution would be within the error of the measurement.

It would be desirable then to obtain an independent value of *μ* as a further check. The method that appeared most feasible for this was to determine the shear modulus, *G*, from the torsional resonance frequency and then, since *E* is already known, to compute *μ* from the well-known relation between *E* and *G* for isotropic materials,

$$\mu =\frac{E}{2G}-1.$$ (6)

For the specimens of set II, *G* is already known from the previous investigation [1] to be 822.1 kilobars. For the specimens of set I, however, it was not possible (at first) to detect the torsional resonance frequency of the round bars by any of the variations of the method previously described.^2^ To circumvent this difficulty, three of the specimens were machined to square cross section. This was in fact the original reason for squaring some of the round specimens of set I. (These squared bars incidentally provided additional specimens for which longitudinal and flexural resonance frequencies could be determined. It can be seen from table 1 that for specimens of this size, the effect of cross section in reducing the rod velocity is of the same order of magnitude as for circular cross section.) For square specimens the torsional resonance frequency, and hence *G*, can be obtained in the manner described in the previous paper [1].

Two of the longer and one relatively short specimen were selected. A square rather than rectangular cross section was chosen because the shape factor for square cross section is believed to be more accurate [1] and would therefore lead to a more accurate value of *G.*

The value of *G* obtained for specimens I–12a, I–15a, and I–18a, of set I, were 820.5, 821.6, 820.5 respectively, with an average of 820.9 kilobars.

Substituting the known values of *E* and *G* for both sets of specimens in eq (6) yields the following values for *μ*: For set I, *μ*=0.269 and for set II, *μ* = 0.285.

The physical constants obtained for sets I and II are now summarized in table 4.

It appears far more likely that the value of *μ* = 0.292 is closer to the true value for both sets of specimens than the values obtained from eq (6). This belief is supported by the following evidence.

The value of *μ* for steel usually found in the literature is around 0.29. Markham [6], for instance, measured *E* and *G* for 10 different types of steel by an ultrasonic method and, from these elastic moduli, calculated *μ*. His values for *μ* varied between 0.286 and 0.292 with an average of 0.289. Analysis of Markham’s data shows that the variation in values of *μ* given for the different types of steel can be accounted for completely by precision in measurements of *E* and *G*, given by Markham, rather than by any differences in the values of *E* and *G* themselves. Therefore, the average value of *μ*=0.289 for all the steels may be taken as characteristic of each of them. Thus it appears that though *E* and *G* may be different for different types of steel, these elastic moduli vary concomitantly so that *μ* remains constant.

If *μ*≈0.292 is correct for the specimens of sets I and II, then a possible explanation for the lower values of *μ* obtained from eq (6) lies in the fact that a preferred crystal orientation develops in the steel during the process of manufacture. Consequently, the assumption of macroscopic isotropy resulting from a completely random crystal orientation is not entirely fulfilled, and eq (6) which is based on this assumption, is not entirely valid for these specimens. Frankland and Whittemore [7] have demonstrated that the average *E* for specimens of “black” sheet steel cut perpendicular to the direction of rolling is significantly different from the average *E* of specimens cut parallel to the direction of rolling. In this connection, it is noteworthy that for the specimens of set I, in which the process of repeated cold working of the parent stock results in a more pronounced crystal orientation, the value of *μ* departs by a greater amount from the “correct” value, than for the specimens of set II, where the process of annealing of the parent stock largely restores the random crystal orientation. Indeed, the value of *μ* in the specimens of set II from eq (6) is in good agreement with the values found in the literature and with that based on Bancroft in this investigation.

Also, it appears from the fact that the empirical points of *v/v*_0_ for sets I and II lie on the same line, that the value of *μ* for both sets of specimens is the same. This does not prove that the value of *μ* based on Bancroft is “correct” but it does make it improbable that sets I and II should have different values as the results based on eq (6) would indicate, for it would be a most unlikely coincidence that any error resulting from interpolation from Bancroft should lead to the same value of *μ*, if the *μ* of both sets of specimens were actually different. Furthermore, the agreement in *μ* for both sets of specimens is in accordance with Markham’s data.

The alternative possibility to explain the discrepancy in *μ*, is that the values based on eq (6) are correct, and that Bancroft’s correction for cross section and consequently, the value of *μ* based on it are incorrect. Inasmuch as this alternative involves the (at least partial) rejection of Bancroft’s theoretical equation as well as the value of *μ* for steel found in the literature, both widely accepted, its correctness appears most unlikely.

### 3.2. Longitudinal Resonance, Rectangular Specimens

The longitudinal resonance frequencies of the rectangular specimens of both sets are listed in tables 1 and 2 but will not be considered here. It is planned to discuss these in a subsequent paper. It will merely be noted here that specimen II–10 b, having a small nearly square cross section, had a considerably higher resonance frequency (17,100 cps) than the other specimens of the same set. Substituting the resonance frequency and length for this specimen in eq (2) yields a value of *v*=5199 m/sec in agreement with *v*_0_ for this set. This value would be expected for a round specimen of the same *k*/*l*.

### 3.3. Flexural Vibrations, Sets I and II

Flexural vibrations are probably of more practical importance than longitudinal as a means of determining Young’s modulus because flexural vibrations can usually be excited more easily than longitudinal. This is especially true for thin specimens. Thus, for these thin specimens where any error in *E* due to an error in the correction for cross section would be minimized, the longitudinal resonance frequency is relatively difficult to obtain, whereas the flexural resonance frequency becomes experimentally easier to excite. Hence a reliable relationship from which *E* may be determined from the flexural resonance frequency becomes important.

Hudson’s [4] numerical solution of the Pochhammer-Chree equations for flexural waves has already been mentioned. Unfortunately, no comparison can be made between Hudson’s results and the empirical ones, because no simple or clear-cut relation has been found to exist between the length of a traveling flexural wave in a very long bar and the length of bars vibrating in flexural resonance. Consequently, one relies on a direct relation between Young’s modulus and the flexural resonance frequency.

Goens [8] has solved Timoshenko’s [9] equation relating Young’s modulus to the flexural resonance frequency for bars of different cross section. Pickett [10] has further simplified Goen’s solution. Goen’s solution can be expressed in the following form:

$$E={\left[\frac{2\pi l^{2}f}{km^{2}}\right]}^{2}\rho T$$ (7)

where *f*, in this case, is the flexural resonance frequency; *k* is the radius of gyration of the cross sectional area about an axis perpendicular to the plane of vibration. For a rectangular cross section $k=t/\sqrt{12}$, *t* being the dimension in the direction of vibration. (The depth and width interchange as *t* depending on whether the vibration is flatwise or edgewise.) For a circular cross section, *k=r*/2; *m* is a constant which has higher values for higher overtones, for the fundamental *m*=4.730; *T* is a correction factor which varies with *k*/*l* and *μ.* Pickett used subscripts for *m* and *T* since both factors vary with the order of vibrations. Since only the fundamental flexural resonance frequency is considered here, the subscripts are dropped.

For cylindrical bars eq (7) becomes

$$E=1.2619{\left[\frac{l^{2}f}{D}\right]}^{2}\rho T,$$ (7a)

and for rectangular bars eq (7) becomes

$$E=0.9464{\left[\frac{l^{2}f}{t}\right]}^{2}\rho T.$$ (7b)

**Figure 2:**
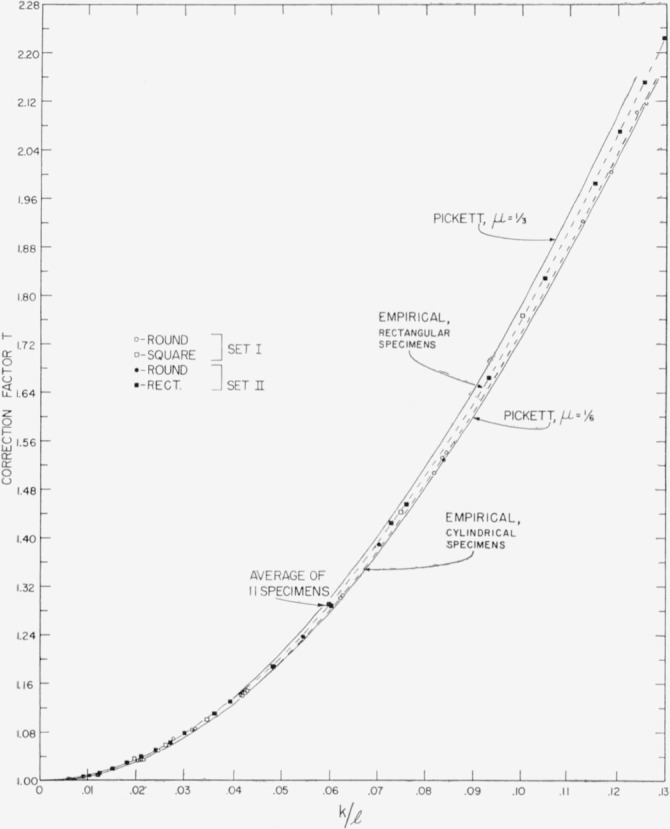
Empirical and theoretical curves showing the effect of *k/l* on the correction factor, *T*, for flexural vibrations. *k/l* is the ratio of the radius of gyration of the cross sectional area about an axis perpendicular to the plane of vibration to the length of the specimen. Sets I and II represent two separate sets of steel bars.

Pickett gives algebraic equations relating *T* to *k*/*l* for *μ*=0, ⅙, and ⅓. In addition he gives numerïcal solutions of these equations for particular values of *k*/*l* and graphs based on these solutions. The graphs for *μ*=⅙ and ⅓, the two values which span the range of interest for steel, are reproduced in figure 2 from Pickett’s numerical values. *T* approaches 1 as *k*/*l* approaches 0 for all values of *μ*.

According to eq 7b, for a given value of *E*, the flexural resonance frequencies of rectangular specimens are independent of the dimension perpendicular to the plane of vibration. Pickett shows in the appendix of his paper, which deals with the problem more rigorously, that in the extreme cases of an infinitely thin bar or an infinitely wide slab, this dimension (perpendicular to the plane of vibration) does slightly affect the flexural resonance frequency. However, for the specimens used in this investigation, this correction would be insignificant.

This means that if the specimens of set II are really uniform with respect to *E* as well as *ρ*, then the edgewise flexural resonance frequency of specimens II–2 through II–12 should all be equal, since the only variable for these specimens is the dimension perpendicular to the plane of vibration. The degree of agreement in this frequency is a critical indication of the intrinsic uniformity of the specimens. The variation in frequency is insignificantly small, as shown in table 3. Therefore, the specimens must be uniform with respect to *E* as well as *ρ*. The importance of this result can hardly be overemphasized, since the uniformity of the specimens with respect to *E* and *ρ* is the foundation of the entire empirical approach.

For the specimens of set I no such conclusive check on the uniformity of *E* is possible, so that the uniformity of *ρ* must serve as indirect evidence of the uniformity of *E.* However, the evidence just presented for the specimens of set II makes it more likely that the same situation holds for the specimens of set I.

The empirical values of *T* are obtained by substituting the base value of *E* for each set of specimens, given in table 4, and the other appropriate parameters for each specimen, all of which are known from tables 1 and 2, in eq 7a or 7b.

It is interesting to compare the values of *E* which result from a determination based on eq 7a and 7b, using *T* obtained directly from Pickett, with the base values of *E* used above. For this purpose, only those specimens of each set are used which have the lowest values of *k*/*l* because for these, as was the case for longitudinal vibrations, it can be seen from the theoretical curves in figure 2 that an error in the choice of *μ* would cause only a negligible error in *T.* Values of *E* for these specimens of low *k*/*l* are given below:

Set I, average of specimens I–19, I–20, I–21, and I–22—2085 kilobars;
Set II, average of specimens II–4r_1_ and II–4r_2_— 2113 kilobars;
Set II, average of specimens II–11 and II–12—2115 kilobars.

These values are seen to be in excellent agreement with those based on longitudinal vibrations and given in table 4. The values based on longitudinal vibrations are used in establishing the empirical values of *T* because the equations on which they are based are established by long usage.

**Figure 3:**
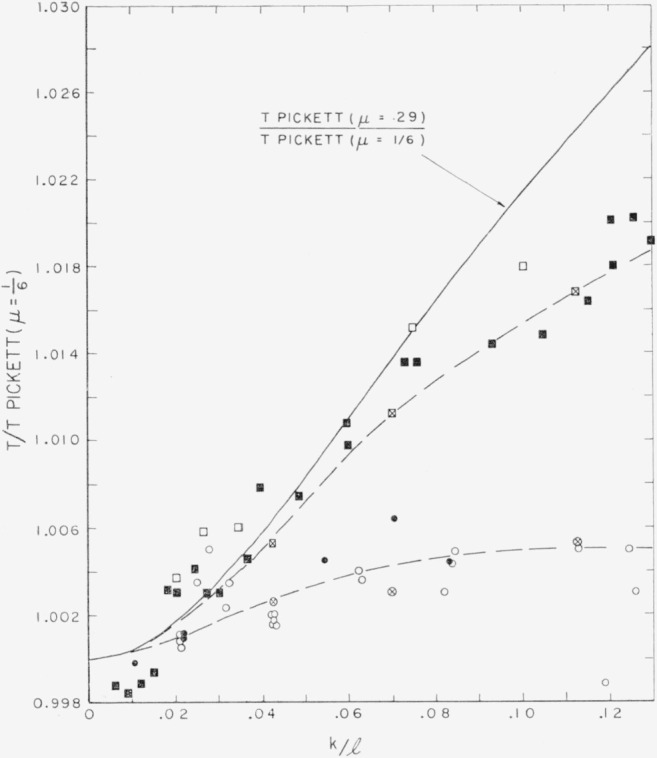
Expanded method of showing data in figure 2 illustrating (a), separation of empirical correction factor for round and rectangular specimens, and (b), departure of all empirical points from theoretical (solid) curve for μ = 0.29. Square symbols represent rectangular specimens; round symbols represent cylindrical specimens; hollow symbols, set I; solid symbols, set II; symbols with crosses, special group (footnote 4).

The empirical values of *T* are given numerically in tables 1 and 2 and are plotted as a function of *k*/*l* in figure 2. The average value of *T* obtained from the edgewise flexural resonance frequency for specimens II–2 through II–12 of set II provide a single point which is designated in the figure. Figure 3 shows the same data in an expanded form.

The values for *T* from Pickett, for *μ*=0.29 given in figure 3, were obtained by a quadratic interpolation from Pickett’s equations for *μ*=0, 1/6, and 1/3.^3^

The computations involved in obtaining *T* for a given value of *μ* from this equation are obviously more cumbersome than from the corresponding one given in the ASTM Book of Standards, pt. 3, p. 1355, 1955 (C215–55T). However, the equation given in the ASTM is inadequate necessitating the use of the equation given here.

Inspection of figures 2 and 3 shows the empirical points to fall on two distinctly separate curves. The points determining these curves are not grouped on the basis of which set of specimens they are comprised but rather on the basis of whether the specimens are cylindrical or rectangular. All of the points forming the upper curve are derived from the rectangular specimens of sets I and II, while all of the points forming the lower curve are derived from the cylindrical specimens of both sets.

Inasmuch as the empirical curves are developed without any assumption for the value of *μ*, these data support the conclusion drawn previously from longitudinal vibrations; namely, that the specimens of both sets have the same value of *μ*.

However, the separation of the empirical points into two curves, one for cylindrical and one for rectangular specimens, is unexpected; for, according to Pickett, the value of *T* at any given *k*/*l* should depend only upon *μ* and not upon whether the bars are circular or rectangular in cross section.^4^ Actually, Pickett recognizes that an assumption is involved in the equality of *T* for specimens of circular and square cross section.

Since the two empirical curves do diverge, especially at higher values of *k*/*l*, it is relevant to inquire which empirical curve is in better agreement with Pickett’s theoretical relation. An estimate of the probable values of *μ* for the two curves may be made on the basis of their relative positions from Pickett’s curves for *μ*=⅓ and *μ*=⅙. Such an estimate leads to a value of *μ* for the upper curve of around 0.26 to 0.30 while for the lower curve *μ* appears to be around 0.17 to 0.19. Inasmuch as the *μ* value so estimated for the upper curve is in agreement with the literature as well as earlier parts of this investigation, while the similar estimate for the lower curve leads to an absurdly low value of *μ* for steel, one concludes that the empirical curve for rectangular specimens is in better agreement with Pickett than the empirical curve for cylindrical specimens. It also appears that Pickett’s curve for *μ* = ⅙ would give reasonably good values of *T* for cylindrical specimens having an actual value of *μ*≈0.29.

Inspection of figure 3 also shows that the empirical curve for rectangular specimens departs from the theoretical curve for *μ*=⅙ by an increasing amount as *k*/*l* increases. For the cylindrical specimens the curve appears to level off to a value slightly above Pickett’s curve for *μ*=⅙.

## 4. Summary

1. Empirical relations have been developed from which Young’s modulus may be determined from the longitudinal and flexural resonance frequencies. Two sets of steel bars were used as specimens. Both sets were composed of cylindrical and rectangular specimens. These empirical relations have been compared with corresponding theoretical ones. The accuracy of the empirical determinations are such that numerical comparisons with the theory to four significant figures are justified.
2. For longitudinal vibrations, the empirically determined curve for the cylindrical specimens, agrees with the corresponding theoretical one (based on Bancroft’s numerical solution of the Pochhammer-Chree equations for this particular boundary condition) if a value of Poisson’s ratio of 0.292 is assumed for both sets of specimens. This value is in agreement with that found in the literature for steel.
3. For flexural vibrations two separate empirical curves develop. One curve is formed by the rectangular specimens of both sets and a second curve is formed by the cylindrical specimens of both sets. The curve formed by the rectangular specimens is in fair agreement with the corresponding theoretical relation (based on Timoshenko, Goens, and Pickett) if a value of Poisson’s ratio about 0.292 is again assumed. However, the empirical curve formed by the cylindrical specimens would agree with the theoretical one only if a Poisson’s ratio of about ⅙ is assumed for them. Since this value is obviously too low for steel, based on the literature and the present study, it is concluded that the experimental results agree with the theory for rectangular specimens but that Pickett’s equations give too high a value for the correction factor for cylindrical specimens.

## Figures and Tables

**Table 1:** Data for specimens of set I^a^

Specimen	Length *l*	Density *ρ*	*f*(long)^b^	*f*(flex)^b^	*k/l*^c^	*υ/υ*_0_ empirical	*T* empirical
							
	*cm*	*g/cm*^3^	*cps*	*cps*			
I–1	5.050	7.849	50,253	31,310	0.12565	0.9851	2.1234
I–2	5.118	7.850	49,606	30,650	.12398	.9857	2.0997
I–3	5.354	7.849	……	28,624	.11850	……	1.9998
I–4	5.639	7.850	……	26,401	.11251	……	1.9206
I–5	7.511	7.851	34,051	16,605	.08447	.9929	1.5424
I–6	7.584	7.851	33,752	16,341	.08366	.9938	1.5320
I–7	7.760	7.851	32,975	15,733	.08176	.9934	1.5077
I–8	10.117	7.847	25,370	9,939	.06272	.9965	1.3085
I–9	10.224	7.846	25,107	9,755	.06206	.9966	1.3024
I–10	14.790	7.851	17,389	4,965	.04289	.9985	1.1475
I–11	14.958	7.853	17,190	4,859	.04241	.9982	1.1447
I–12	14.968	7.854	17,185	4,854	.04238	.9986	1.1438
I–13	15.118	7.853	17,014	4,765	.04196	.9985	1.1410
I–14	15.235	7.851	16,883	4,695	.04164	.9985	1.1395
I–15	19.736	7.851	13,037	2,866	.03214	.9989	1.0862
I–16	19.985	7.852	12,875	2,799	.03174	.9989	1.0829
I–17	22.951	7.849	11,213	2,139	.02766	.9991	1.0664
I–18	25.641	7.850	10,039	1,725	.02475	.9993	1.0525
I–19	29.987	7.852	8,587	1,271	.02116	.9997	1.0360
I–20	30.201	7.853	8,525	1,253	.02101	.9995	1.0359
I–21	30.500	7.852	8,442	1,229	.02080	.9998	1.0353
I–22	30.554	7.850	8,427	1,225	.02076	.9996	1.0350
I-12b	5.177	7.849	……	26,704	.10020	……	1.7649
I–12c	6.942	7.849	……	16,415	.07473	……	1.4445
I–12a	14.968	7.850	17,188	4,043	.03464	.9988	1.1016
I–15a	19.736	7.848	13,039	2,370	.02627	.9991	1.0610
I–18a	25.641	7.847	10,038	1,421	.02023	.9992	1.0360

a All specimens except those followed by a letter are 2.5378 cm in diameter. Those followed by a letter are 1.796-cm square.

b Fundamental longitudinal and flexural resonance frequencies.

c *k*=radius of gyration of the cross sectional area about an axis perpendicular to the plane of vibration
for round specimens *k*=¼ diam=0.63445 cm. for square specimens $k=\text{edge}/\sqrt{12}=0.51846$ cm. for cylindrical specimens, *D*/λ=2*k/l* where D=diameter of specimen and λ=wavelength of longitudinal wave.

**Table 2:** Data for specimens of set II

Specimen^a^	Rectangular specimens										
	Length *l*	Width *w*	Depth *d*	Density *ρ*	Flatwise			Edgewise			*f* (long)
					*k/l*^b^	*f*(flex)	*T*	*k/l*^b^	*f*(flex)	*T*	
											
	*cm*	*cm*	*cm*	*g/cm*^3^		*cps*			*cps*		*cps*
II-I	15.202	3.1496	3.1496	7.817	0.05981	6411	1.2915	0.05981	6411	1.2915	17,046
II-2	15.202	3.1433	2.5405	7.819	.04825	5379	1.1936	.05969	6399	1.2910	17,053
II-3	15.202	3.1433	1.9055	7.814	.03619	4183	1.1112	.05969	6398	1.2923	17,062
II-4	15.202	3.1433	1.5875	7.817	.03015	3538	1.0775	.05969	6400	1.2909	17,065
II-5	15.202	3.1433	1.4300	7.814	.02716	3208	1.0635	.05969	6397	1.2926	17,066
II-6	15.202	3.1433	1.2708	7.816	.02413	2867	1.0514	.05969	6399	1.2915	17,066
II-7	15.202	3.1433	1.1120	7.812	.02112	2523	1.0403	.05969	6399	1.2921	17,071
II-8	15.202	3.1433	0.9530	7.812	.01810	2172	1.0309	.05969	6394	1.2941	17,067
II-9	15.202	3.1433	.7943	7.813	.01508	1820.6	1.0189	.05969	6400	1.2916	17,075
II-10	15.202	3.1433	.6363	7.815	.01208	1463.4	1.0118	.05969	6400	1.2912	17,070
II-II	15.202	3.1433	.4773	7.811	.00906	1101.1	1.0061	.05969	6400	1.2919	17,070
II-12	15.202	3.1433	.3172	7.814	.00602	733.0	1.0028	.05969	6396	1.2927	17,064
II-2a	7.882	3.1433	2.5405	7.814	.09306	16,941	1.6660	.11513	19,203	1.9849	……
II-2b	7.010	3.1433	2.5405	7.817	.10463	20,432	1.8297	.12945	22,934	2.2232	……
II-3a	7.546	3.1433	1.9055	7.818	.07290	14,983	1.4255	.12026	20,495	2.0732	……
II-3b	7.249	3.1433	1.9055	7.817	.07589	16,052	1.4584	.12519	21,789	2.1535	……
II-10a	15.202	2.0574	0.6363	7.816	.01208	1460.7	1.0147	.03907	4475	1.1314	……
II-10b	15.202	0.6426	.6363	7.814	.01220	……	……	……	……	……	17,100
	

	Cylindrical specimens							
	Length *l*	Diameter *D*	Density *ρ*	*k/l*^c^	*f*(flex)	*T*	*f*(long)	*υ/υ*_0_
								
II-1*r*	14.364	3.1252	7.816	0.05439	6303	1.2384	18, 057	0.9978
II-2*ar*	7.315	2.4460	7.812	.08359	17,102	1.5329	35, 329	.9944
II-3*br*	6.447	1.8124	7.812	.07029	17,141	1.3882	40,145	.9957
II-4*r*_1_	14.669	1.2845	7.816	.02189	2708.9	1.0404	17, 714	.9996
I1-4*r*_2_	14.668	1.2843	7.816	.02189	2708.3	1.0407	17, 715	.9996

a Letter following specimen number indicates that the specimen has been redimensioned. Number denotes original specimen. A second letter indicates a second change in dimension.

b *k*=radius of gyration of the cross sectional area about an axis perpendicular to the plane of vibration. For rectangular specimens in flatwise vibrations, $k=d/\sqrt{12}$; for edgewise vibration $k=w\;\sqrt{12}$.

c For cylindrical specimens, *k*=D/4. Since, for the fundamental longitudinal resonance frequency, λ=2*l, k/l*=*d*/2λ.

**Table 3:** Overtones of logitudinal resonance vibrations of several cylindrical specimens

	Frequency	*D*/λ=*r/l*	*υ/υ*_0_^a^
$\begin{array}{cc} \begin{array}{c} \text{Set}\;I\\ \text{Specimen I}-20 \end{array} & \{\begin{array}{l} \text{Fundamental}\;(n=1)\\ 1\text{st overtone}\;(n=2)\\ 2\text{nd overtone}\;(n=3)\\ 3\text{rd overtone}\;(n=4)\\ 4\text{th overtone}\;(n=5) \end{array} \end{array}$	8525	0.04202	0.9995
	17027	.08405	.9982
	25491	.12607	.9963
	33898	.16809	.9936
	42218	.21012	.9900
$\begin{array}{cc} \begin{array}{c} \text{Set}\;I\\ \text{Specimen I}-21 \end{array} & \{\begin{array}{l} \text{Fundamental}\;(n=1)\\ 1\text{st overtone}\;(n=2)\\ 2\text{nd overtone}\;(n=3)\\ 3\text{rd overtone}\;(n=4)\\ 4\text{th overtone}\;(n=5) \end{array} \end{array}$	8442	.04160	.9996
	16858	.08321	.9981
	25238	.12481	.9961
	33560	.16642	.9935
	41800	.20802	.8999
$\begin{array}{cc} \begin{array}{c} \text{Set}\;I\\ \text{Specimen I}-19 \end{array} & \{\begin{array}{l} \text{Fundamental}\;(n=1)\\ 1\text{st overtone}\;(n=2)\\ 2\text{nd overtone}\;(n=3)\\ 3\text{rd overtone}\;(n=4) \end{array} \end{array}$	8587	.04232	.9997
	17154	.08464	.9985
	25683	.12696	.9966
	34146	.16928	.9938
$\begin{array}{cc} \begin{array}{c} \text{Set}\;I\\ \text{Specimen I}-16 \end{array} & \{\begin{array}{l} \text{Fundamental}\;(n=1)\\ 1\text{st overtone}\;(n=2) \end{array} \end{array}$	12875	.06348	.9989
	25692	.12696	.9967
$\begin{array}{cc} \begin{array}{c} \text{Set}\;\mathrm{II}\\ \text{Specimen II}-1r \end{array} & \{\begin{array}{l} \text{Fundamental}\;(n=1)\\ 1\text{st overtone}\;(n=2)\\ 2\text{nd overtone}\;(n=3) \end{array} \end{array}$	18057	.10878	.9978
	35823	.21756	.9898
	52884	.32634	.9741

a For set I specimens, *υ*_0_=5152 m/sec; for set II specimens, *υ*_0_=5199 m/sec.

**Table 4:** Physical constants of two different sets of steel specimens

	Set I	Set II
*υ*_0_, “rod velocity” m/sec	5152	6199
*ρ*, density g/cm^3^	7.851	7.816
*E*, Young’s modulus kilobars	2084	2113
*G*, shear modulus kilobars	820.9	822.1
*μ*, Poisson’s ratio	0.269^a^	0.285^a^

a Derived from *E* and *G* values and eq (6). From Bancroft, *μ*=0.292 for both sets.

## Footnotes

1 Figures in brackets indicate the literature references at the end of this paper.

2 Subsequently, by the use of an improved driver and suspension of the specimens from strings as already described, the torsional resonance frequencies were obtained for cylindrical bars of sets I and II. The average values of *G*, calculated from these resonances, were in agreement with those given in the text.

3 The actual equation used for this interpolation was:

$$\begin{array}{l} T=1+79.02(1+0.0752\;\mu +0.8109\;\mu^{2})\;{\left(k/l\right)}^{2}\\ \;-125{\left(\frac{k}{l}\right)}^{4}-\frac{1201(1+0.2023\;\mu +2.173\;\mu^{2})\;{\left(k/l\right)}^{4}}{1+76.06(1+0.14081\;\mu +1.536\;\mu^{2})\;{\left(k/l\right)}^{2}}. \end{array}$$

4 This unexpected result was further tested using an entirely different group of six specimens all cut from the same soft steel. Special care was taken to have the specimens homogeneous and isotropic. The six specimens were divided into three pairs, each pair having the same length and the same value of *k.* One specimen of each pair was circular and the other square in cross section. If Pickett’s assumption of the equality of the correction factor *T* for square and cylindrical bars of the same *k/l* is correct, then each of the above pairs should have the same flexural resonance frequency. However, the frequencies of each pair were found to differ from each other by an amount in agreement with the empirical results already obtained for sets I and II. Points representing these specimens are included in figure 3.

## References

[1] Spinner S, Valore RC (1958). Comparison of theoretical and empirical relations between the shear modulus and torsional resonance frequencies for bars of rectangular cross section. J Research NBS.

[2] Kolsky H (1953). Stress waves in solids, Preface.

[3] Bancroft D (1941). The velocity of longitudinal waves in cylindrical bars. Phys Rev.

[4] Hudson GE (1943). Dispersion of elastic waves in solid circular cylinders. Phys Rev.

[5] Davies RM, Batchelor GK, Davies RM (1956). Stress waves in solids, Surveys in Mechanics.

[6] Markham MF (1957). Measurement of elastic constants by the ultrasonic pulse method. Brit J Appl Phys.

[7] Frankland JM, Whittemore HL (1932). Tests of cellular sheet steel flooring. J Research NBS.

[8] Goens E (1931). Uber die Bestimmung des Elastizitatsmoduls von Staben mit Hilde von Biegung Schwingungen. Ann der Phys, B Folge.

[9] Timoshenko SP (1922). On the transverse vibrations of bars of uniform cross section. Phil Mag Ser 6.

[10] Pickett G (1945). Equations for computing elastic constants from flexural and torsional resonant frequencies of vibration of prisms and cylinders. Proc ASTM.

